# Multisectoral interventions and health system performance: a systematic review

**DOI:** 10.2471/BLT.23.291246

**Published:** 2024-04-30

**Authors:** I Nyoman Sutarsa, Lachlan Campbell, I Made Dwi Ariawan, Rosny Kasim, Robert Marten, Dheepa Rajan, Sally Hall Dykgraaf

**Affiliations:** a School of Medicine and Psychology, College of Health and Medicine, Australian National University, 54 Mills Road, Acton 2601, Australia.; b Department of Public Health and Preventive Medicine, Faculty of Medicine, Udayana University, Indonesia.; c European Observatory on Health Systems and Policies, Brussels, Belgium.

## Abstract

**Objective:**

To conduct a systematic review on the effects of multisectoral interventions for health on health system performance.

**Methods:**

We conducted a systematic review according to the preferred reporting items for systematic review and meta-analysis protocols. We searched for peer-reviewed journal articles in PubMed®, Scopus, Web of Science, Cumulated Index to Nursing and Allied Health Literature, and the Cochrane Database of Systematic Reviews on 31 August 2023 (updating on 28 February 2024). We removed duplicates, screened titles and abstracts, and then conducted a full-text eligibility and quality assessment.

**Findings:**

We identified an initial 1118 non-duplicate publications, 62 of which met our inclusion and exclusion criteria. The largest proportions of reviewed studies focused on multisectoral interventions directly related to specific health outcomes (66.1%; 41 studies) and/or social determinants of health (48.4%; 30 studies), but without explicit reference to overall health system performance. Most reviewed publications did not address process indicators (83.9%; 52/62) or discuss sustainability for multisectoral interventions in health (72.6%; 45/62). However, we observed that the greatest proportion (66.1%; 41/62) considered health system goals: health equity (68.3%; 28/41) and health outcomes (63.4%; 26/41). Although the greatest proportion (64.5%; 40/62) proposed mechanisms explaining how multisectoral interventions for health could lead to the intended outcomes, none used realistic evaluations to assess these.

**Conclusion:**

Our review has established that multisectoral interventions influence health system performance through immediate improvements in service delivery efficiency, readiness, acceptability and affordability. The interconnectedness of these effects demonstrates their role in addressing the complexities of modern health care.

## Résumé

**Objectif:**

Réaliser une revue systématique consacrée à l'impact des interventions multisectorielles sur la performance des systèmes de santé.

**Méthodes:**

Nous avons procédé à une revue systématique en appliquant les éléments de rapport privilégiés dans les protocoles de revues systématiques et méta-analyses. Nous avons exploré PubMed®, Scopus, Web of Science, Cumulated Index to Nursing and Allied Health Literature, ainsi que la Base de données Cochrane des revues systématiques le 31 août 2023 (mise à jour le 28 février 2024), à la recherche d'articles de revue évalués par des pairs. Ensuite, nous avons supprimé les doublons, passé les titres et résumés au crible, puis déterminé la qualité et l'admissibilité des articles complets.

**Résultats:**

Nous avons initialement identifié 1118 publications non dupliquées; 62 d'entre elles répondaient à nos critères d'inclusion et d'exclusion. Une grande partie des études examinées portaient sur des interventions multisectorielles en lien direct avec des résultats de santé spécifiques (66,1%; 41 études) et/ou des déterminants sociaux de la santé (48,4%; 30 études), sans toutefois faire explicitement référence à la performance globale des systèmes de santé. La majorité des publications ne mentionnaient aucun indicateur de processus (83,9%; 52/62) et n'abordaient pas la durabilité des interventions multisectorielles dans le domaine de la santé (72,6%; 45/62). Nous avons néanmoins constaté qu'en général, elles tenaient compte des objectifs relatifs aux systèmes de santé (66,1%; 41/62): l'équité en santé (68,3%; 28/41) et les résultats de santé (63,4%; 26/41). Bien que la plupart (64,5%; 40/62) proposent des mécanismes visant à expliquer comment les interventions multisectorielles en matière de santé pourraient amener aux résultats escomptés, aucune n'avait recours à des évaluations réalistes pour les mesurer.

**Conclusion:**

Notre revue nous a permis d'établir que les interventions multisectorielles influençaient la performance des systèmes de santé à travers des améliorations immédiates en termes d'efficacité, de disponibilité, d'acceptation et d'abordabilité des prestations de services. L'interdépendance entre ces effets témoigne de l'importance qu'ils revêtent lorsqu'il s'agit d'appréhender les rouages complexes des soins de santé modernes.

## Resumen

**Objetivo:**

Realizar una revisión sistemática sobre los efectos de las intervenciones multisectoriales en favor de la salud sobre el rendimiento de los sistemas sanitarios.

**Métodos:**

Se realizó una revisión sistemática de acuerdo con los ítems de informe preferidos para los protocolos de revisión sistemática y metanálisis. Se realizaron búsquedas de artículos de revistas con revisión por pares en PubMed®, Scopus, Web of Science, Cumulated Index to Nursing and Allied Health Literature y la Base de Datos Cochrane de Revisiones Sistemáticas el 31 de agosto de 2023 (actualización el 28 de febrero de 2024). Se eliminaron los duplicados, se examinaron los títulos y los resúmenes y, a continuación, se realizó una evaluación de la elegibilidad y la calidad del texto completo.

**Resultados:**

Se identificaron 1118 publicaciones iniciales no duplicadas, 62 de las cuales cumplían los criterios de inclusión y exclusión. El mayor porcentaje de estudios revisados se centró en intervenciones multisectoriales directamente relacionadas con resultados sanitarios específicos (66,1%; 41 estudios) o determinantes sociales de la salud (48,4%; 30 estudios), pero sin referencia explícita al rendimiento general del sistema sanitario. La mayoría de las publicaciones revisadas no abordaron indicadores de proceso (83,9%; 52/62) ni discutieron la sostenibilidad de las intervenciones multisectoriales en salud (72,6%; 45/62). Sin embargo, se observó que el mayor porcentaje (66,1%; 41/62) tenía en cuenta los objetivos del sistema sanitario: equidad sanitaria (68,3%; 28/41) y resultados sanitarios (63,4%; 26/41). Aunque el mayor porcentaje (64,5%; 40/62) propuso mecanismos que explicaban cómo las intervenciones multisectoriales para la salud podían conseguir los resultados previstos, ninguno empleó evaluaciones realistas para evaluarlos.

**Conclusión:**

La revisión que se realizó ha demostrado que las intervenciones multisectoriales influyen en el rendimiento de los sistemas sanitarios a través de mejoras inmediatas en la eficiencia, la disponibilidad, la aceptabilidad y la asequibilidad de la prestación de servicios. La interconexión de estos efectos demuestra su función a la hora de abordar las complejidades de la atención sanitaria moderna.

## ملخص

الغرض إجراء مراجعة منهجية على آثار التدخلات الصحية متعددة القطاعات على أداء النظام الصحي.

الطريقة قمنا بإجراء مراجعة منهجية وفقاً للعناصر المفضلة لإعداد التقارير لدى بروتوكولات المراجعة المنهجية والتحليل التلوي. قمنا بالبحث عن مقالات صحفية تمت مراجعتها بواسطة الأقران في قواعد البيانات PubMed®، وScopus، وWeb of Science، وCumulated Index to Nursing (الفهرس التراكمي للتمريض)، وAllied Health Literature (المؤلفات الصحية المساعدة)، وقاعدة بيانات Cochrane للمراجعات المنهجية، في 31 أغسطس/آب 2023 (تم تحديثها في 28 فبراير/شباط 2024). وقمنا بإزالة التكرارات، والعناوين والملخصات التي تم فحصها، ثم أجرينا تقييمًا لجودة النص الكامل ومدى توفر الشروط فيه.

النتائج قمنا بتحديد 1118 منشوراً أولياً غير مكرر، استوفت 62 منها معايير الإدراج والاستبعاد لدينا. ركزت النسب الأكبر من الدراسات التي تمت مراجعتها على التدخلات متعددة القطاعات المرتبطة بشكل مباشر بنتائج صحية محددة (%66.1؛ 41 دراسة) و/أو المحددات الاجتماعية للصحة (%48.4؛ 30 دراسة)، ولكن دون إشارة صريحة إلى الأداء العام للنظام الصحي. لم تتناول معظم المنشورات التي تمت مراجعتها مؤشرات العملية (%83.9؛ 52/62)، ولم تناقش استدامة التدخلات متعددة القطاعات في الصحة (%72.6؛ 45/62). ومع ذلك، فقد لاحظنا أن النسبة الأكبر (%66.1؛ 41/62) وضعت في اعتبارها أهداف النظام الصحي: العدالة الصحية (%68.3؛ 28/41) والنتائج الصحية (%63.4؛ 26/41). وعلى الرغم من أن النسبة الأكبر (%64.5؛ 40/62) قد اقترحت آليات تشرح كيف يمكن للتدخلات الصحية متعددة القطاعات أن تؤدي إلى النتائج المنشودة، إلا أنه لم يكن من بين هذه الآليات ما يعتمد على التقييمات الواقعية لتقييم هذه النتائج.

الاستنتاج أثبتت المراجعة التي قمنا بها أن التدخلات متعددة القطاعات تؤثر على أداء النظام الصحي من خلال التحسينات الفورية في كفاءة تقديم الخدمة، ودرجة الاستعداد، ودرجة القبول، وإمكانية تحمل تكاليفها. إن الترابط الداخلي بين هذه التأثيرات يوضح دورها في معالجة تعقيدات الرعاية الصحية الحديثة.

## 摘要

**目的:**

针对多部门卫生干预措施对卫生系统绩效的影响开展系统评价。

**方法:**

根据适用于系统评价和荟萃分析方法的首选报告项目，我们开展了系统评价。2023 年 8 月 31 日，我们在 PubMed®、斯高帕斯 (Scopus)、Web of Science、护理和联合卫生文献累积索引 (CINAHL) 以及 Cochrane 系统评价数据库中搜索了经同行评审的期刊文章（2024 年 2 月 28 日更新）。我们删除了重复项，筛选了标题和摘要，然后实施了全文合格性和质量评估。

**结果:**

我们初步确定了 1,118 份不重复的期刊文章，其中有 62 份符合我们的纳入和排除标准。在接受系统评价的研究资料中，绝大部分侧重于与特定健康结果直接相关的多部门干预措施（占 66.1%；41 项研究）和/或健康社会决定因素（占 48.4%；30 项研究），但并未明确提及卫生系统的总体绩效。大多数接受系统评价的期刊文章并未提及过程指标（占 83.9%；52/62），或未讨论多部门卫生干预措施的可持续性（占 72.6%；45/62）。但是，据我们观察，绝大部分期刊文章考虑了卫生系统目标（占 66.1%；41/62）：卫生公平（占 68.3%；28/41）和健康结果（占 63.4%；26/41）。尽管绝大部分期文章（占 64.5%；40/62）建议采用解释多部门卫生干预措施如何实现预期结果的机制，但所有文章均未使用现实评估方法来评估这些机制。

**结论:**

通过开展系统评价我们可以确定的是，多部门干预措施可立竿见影地提高服务提供效率、推动准备工作、提高可接受性和可负担性，从而影响卫生系统的绩效。这些影响的相互关联性表明了其在解决现代卫生保健复杂性方面所起的作用。

## Резюме

**Цель:**

Провести систематический обзор влияния межотраслевых мероприятий в области здравоохранения на эффективность системы здравоохранения.

**Методы:**

В соответствии с предпочтительными пунктами отчетности для протоколов систематических обзоров и метаанализов был проведен систематический обзор. По состоянию на 31 августа 2023 года (обновление на 28 февраля 2024 года) был проведен поиск рецензируемых журнальных статей в базах данных PubMed®, Scopus, Web of Science, Cumulated Index to Nursing and Allied Health Literature и Cochrane Database of Systematic Reviews. Были удалены дубликаты, проверены названия и резюме статей, а затем была проведена полнотекстовая оценка приемлемости и качества.

**Результаты:**

Было обнаружено 1118 недублированных публикаций, 62 из которых соответствовали критериям включения и исключения. Наибольшая часть рассмотренных исследований была посвящена межотраслевым мероприятиям, непосредственно связанным с конкретными результатами мероприятий по охране здоровья (66,1%; 41 исследование) и/или социальными детерминантами здоровья (48,4%; 30 исследований), но без прямого указания на общую эффективность системы здравоохранения. В большинстве изученных публикаций не рассматривались показатели процесса (83,9%; 52/62) и не обсуждалась долгосрочная перспектива воздействия межотраслевых мероприятий в сфере здравоохранения (72,6%; 45/62). Однако в наибольшей доле (66,1%; 41/62) из них рассматривались цели системы здравоохранения: обеспечение равенства в вопросах здравоохранения (68,3%; 28/41) и результаты мероприятий по охране здоровья (63,4%; 26/41). Хотя в наибольшей степени (64,5%; 40/62) были предложены механизмы, объясняющие, как межотраслевые мероприятия в сфере здравоохранения могут привести к достижению намеченных результатов, ни в одном из них не использовались реалистичные оценки для их анализа.

**Вывод:**

Результаты обзора свидетельствуют о том, что межотраслевые мероприятия влияют на эффективность системы здравоохранения путем непосредственного повышения эффективности предоставления услуг, готовности, приемлемости и доступности. Взаимосвязь этих эффектов свидетельствует об их роли в решении сложных проблем современного здравоохранения.

## Introduction

There is unequivocal recognition that health and well-being are determined by non-medical factors, including structural, social and commercial determinants of health.^1^ Addressing those determinants is a task for actors both within and outside the health system; creating robust health systems therefore requires health system actors to engage in active collaboration, outreach and partnership with non-health sectors. Such multisectoral collaborations link the health sector with other sectors and entities wielding different forms of influence, such as financial control of integrated budgeting, or educational influences that strengthen community participation and empowerment.

Multisectoral approaches are vital for addressing health issues that extend beyond traditional sectoral boundaries, fostering cross-sectoral accountability and shared responsibility.^2^ These strategies are crucial for achieving equity and the health-related United Nations sustainable development goals (SDGs).^2^^,^^3^

The terms multisectoral and intersectoral are equivalent and frequently used interchangeably, denoting collaborative partnerships across ministries, government agencies, nongovernmental actors and stakeholders with common goals on specific issues. This review focuses on multisectoral action for health, which specifically refers to actions by non-health sectors that address health issues, determinants, equity or protection.^4^^,^^5^ These approaches can occur in collaboration with the health sector, and be either horizontal (between health and non-health actors at the same government level) or vertical (between different government levels). Multisectoral actions are particularly crucial for promoting health amid intersecting economic, social and environmental forces.

Globally, the aim of implementing multisectoral action for health is to leverage health system-strengthening interventions; such interventions would aim to address issues that extend beyond the health system but significantly influence population health and health disparities.^5^^,^^6^ Multisectoral actions are necessary to address some of those influencing factors, including poverty and equity^7^ or zoonotic diseases.^8^ Simultaneously, these approaches can contribute positively to health sector-specific operational issues for addressing complex health problems,^9^^,^^10^ as well as enhance staff satisfaction and professional capacity in primary health care.^2^

Universal health coverage (UHC), a key SDG target, requires strong health systems to provide a broad range of health services, including preventive care and health promotion.^11^ It also needs strong health governance that leverages multisectoral action to enhance access to care, promote health, prevent disease and strengthen community engagement.^2^^,^^9^ For example, health actors’ collaboration with transportation sectors could address accessibility issues by providing transport to health facilities.^12^ Effective synergy between education and health sectors can lead to integration of health promotion into school curriculums, facilitating healthy lifestyles and better long-term health benefits for the population.^13^ Collaboration between finance, social and health sectors may increase investment in health infrastructure and programmes.^14^ Involving various sectors in health planning, implementation and evaluation facilitates resource sharing, including funding and expertise.^14^^,^^15^

Although, to our knowledge, a synthesis of these studies has not been recently undertaken and the impact of multisectoral action on health system performance has not been analysed. 

To synthesize the evidence from previous studies that have examined the effects of multisectoral actions on health system performance, we conducted a systematic review. Findings from this review will provide evidence for policy-makers to design interventions that can translate into improvements in health system performance.

## Methods

### Design and search strategy

Our systematic review adhered to the preferred reporting items for systematic review and meta-analysis protocols.^16^ We listed our review in the International Prospective Register of Systematic Reviews (protocol ID CRD42023438975) on 3 July 2023. For this review, we adopted a broad definition of multisectoral collaboration for health, defined as “actions undertaken by non-health sectors, possibly but not necessarily in collaboration with the health sector, addressing health issues, determinants of health, health equity, or protecting the health of the population.”^5^

We included peer-reviewed journal articles from PubMed®, Scopus, Web of Science, Cumulated Index to Nursing and Allied Health Literature, and the Cochrane Database of Systematic Reviews. We adopted a three-step approach to develop the final search strategies, aiming for a balance between breadth and comprehensiveness. First, we identified articles that represented good examples of multisectoral approaches for health and health system performance, governance and strengthening. These papers were identified through a structured search of the Scopus database and a manual search of cross-references cited in the articles used to prepare the review protocol. This initial step allowed precise development of specific search terms for the review. Searches were conducted with no time or language restrictions across these databases, using search terms outlined in Box 1.

Box 1 **Search strategy for systematic review of the effect of multisectoral interventions for health on health system performance**

Multisectoral OR intersectoral OR multisectorial OR intersectorial OR collaboration OR integration OR partnership* OR coordinat* OR “joined-up” OR synerg* “health in all polic*” OR HiAP OR HEiAP OR “healthy cit*” OR “One Health” OR “healthy public polic*” OR “national health assembly” OR “whole system approach*” OR “whole of government*” OR “whole of city” OR “whole of society” OR “health for all” OR “health in all” OR “health equity in all” OR “health impact assessment” OR HIA OR “system* change” OR “system* transformation” OR “cash transfer”

AND

“health system*” OR “health care” OR “health equity” OR “social determinant* of health” OR “commercial determinant* of health”

AND

efficiency OR responsiveness OR quality OR safety OR “risk protection” OR access* OR equit* OR morbidit* OR mortalit*

AND NOT

“inter-professional” OR “interprofessional”

Second, we searched for peer-reviewed articles from the same databases, applying a combination of keywords and terms that optimized relevant results. The initial searches were performed on 31 August 2023, and an updated search was conducted on 28 February 2024. Our search strategies encompassed all published papers until the end of February 2024. Third, we conducted a manual search of references of included papers to identify any critical additional literature.

### Selection processes

We removed duplicates from search results using EndNote™ Version 20 I(Clarivate, Philadelphia, United States of America) and manually confirmed these removals. We transferred non-duplicate records to Covidence (Veritas Health Innovation, Melbourne, Australia) for screening and data management. We used a two-tiered approach for study selection, involving title and abstract screening and then full-text screening with predetermined inclusion and exclusion criteria. 

Publications were reviewed if they included an assessment of multisectoral or intersectoral collaboration for health on health system performance indicators or on health system strengthening or performance; or if they evaluated the impacts of such collaborations on health systems, equity and health determinants. We considered all study designs, settings and participant types. We excluded publications that focused primarily on interprofessional collaboration in clinical care and telemedicine; that only examined collaborations within the health sector or multisectoral collaborations that did not include the health sector; that did not report any primary data; or were only published in abstract form or in conference proceedings. Two authors independently assessed titles and abstracts, and four authors (two per publication) conducted a full-text review. Disagreements were resolved through consensus and, if needed, a third reviewer.

### Data collection

We extracted review data from included studies using a standardized data charting form, which included bibliographic details, study type, participant information, settings or contexts, collaboration type, evidence of impact, barriers and facilitators for implementation, and proposed mechanisms (online repository).^17^ Four authors undertook data extraction, with each study evaluated by a single author. Discrepancies were resolved through discussion or moderation by a second reviewer. All data were transferred to Excel (Microsoft, Redmond, USA) for further analysis.

### Quality appraisal 

We assessed individual study quality using the mixed methods appraisal tool, version 2018.^18^ We rated each study on a nominal scale (online repository),^19^ providing a descriptive account of the quality of included studies, with difficulties resolved by another reviewer. We used two screening and five methodology questions tailored to the study design to assess the quality of each study; we tabulated assessments and considered these during analysis, interpreting study data carefully while considering any risk of bias.

### Data synthesis

We conducted a narrative synthesis of individual studies to address the review objective, summarizing study and intervention characteristics, reported effects and proposed mechanisms. Because of heterogeneity among the reviewed publications, as well as the complex nature of interventions and broad range of possible effects, we classified and reported intermediate and ultimate effects using tables, narrative descriptions and pooled data when appropriate to present the data.

## Results

**Fig. 1:**
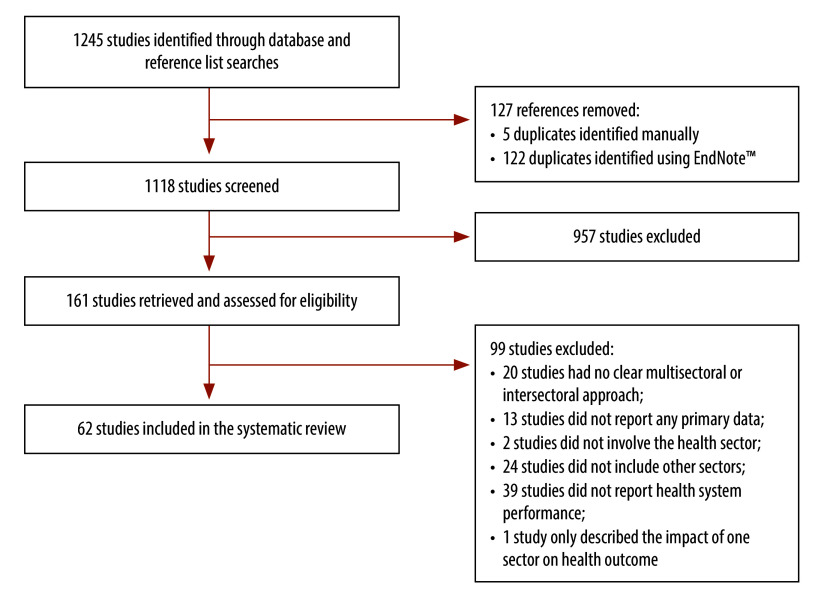
Flowchart of the selection of studies on the effects of multisectoral interventions for health on health system performance

We identified a total of 1118 unique studies and conducted a full-text eligibility assessment of 161 studies. We excluded 99 studies following full-text assessment and based our analysis on the remaining 62 studies (Fig. 1).

We list the characteristics of the 62 reviewed studies^20^^–^^81^ in Table 1 (available at: https://www.who.int/publications/journals/bulletin/) which were conducted in 30 countries across all World Health Organization (WHO) regions (Table 2). Two studies are published in languages other than English: one in Spanish^20^ and one in German.^21^ The publication years of the studies, spanning 2010–2023, indicate an emerging body of evidence.

**Table 1:** Characteristics of the studies included in a systematic review of the effect of multisectoral interventions for health on health system performance

Reference	Country, WHO region, income level^a^	Study objective(s)	Methods	Data analysis
Skeen et al., 2010^71^	South Africa, African Region, upper middle	To assess progress in intersectoral collaboration, and intersectoral roles and responsibilities, for mental health to generate lessons that are potentially applicable to other low- and middle-income countries	Qualitative study using semi-structured interviews, and focus group discussion with policy-makers, health providers, community members and NGOs	Thematic and framework analysis for qualitative data
Barton et al., 2011^66^	European cities, European Region, high	To evaluate progress by European cities in relation to healthy urban planning during Phase IV of WHO’s Healthy Cities programme (2003–2008)	Quantitative study using secondary data analysis	Quantitative analysis using descriptive approach
Paes-Sousa et al., 2011^46^	Brazil, Region of the Americas, upper middle	To identify potential associations between enrolment in the *Programa Bolsa Família* and the anthropometric indicators: height for age, weight for age and weight for height in children < 5 years	Quantitative cohort study using secondary data analysis	Quantitative analysis using predictive or modelling analysis
Storm et al., 2011^25^	Netherlands (Kingdom of the), European Region, high	To analyse opportunities to reduce health inequalities in the Dutch population by health in all policies strategy; identify ongoing policy resolution inside and outside the health domain with potential impact on health inequalities (and their determinants); and identify critical factors (e.g. drivers and barriers) with regards to collaboration between various ministries	Qualitative study using policy document review, semi-structured interviews and focus group discussion with policy-makers	Thematic analysis for qualitative data
Ramanadhan et al., 2012^38^	USA, Region of the Americas, high	To explore the concept of community mobilization and intersectoral collaboration in the context of community-based participatory research to reduce cancer disparities	Social network analysis using semi-structured interviews and quantitative survey involving policy-makers, health providers, community members, private sectors, NGOs, media and academics	Quantitative analysis using descriptive, inferential and social networks analysis
Serrate et al., 2012^20^	Cuba, Region of the Americas, upper middle	To identify social actors’ perceptions of the process of intersectoral action, and its implications for population health and well-being	Mixed-methods study using survey (self-administered) and participatory discussion	Qualitative and quantitative descriptive analyses
Fawcett et al., 2013^54^	USA, Region of the Americas, high	To determine whether the implementation of the health for all model (within the Latino Health for All Coalition in Kansas City, Kansas) was consistent with principles of community-based participatory research	Mixed-methods study using semi-structured interviews and quantitative survey with community members and NGOs	Content analysis for qualitative data and descriptive analysis for quantitative data
Guanais, 2013^60^	Brazil, Region of the Americas, upper middle	To examine how enhanced access to medical services and expansion of poverty alleviation measures interact in the reduction of infant mortality	Quantitative study using secondary data analysis for the ecological longitudinal approach	Quantitative analysis using descriptive and inferential analysis
Johnson Thornton et al., 2013^72^	USA, Region of the Americas, high	To describe the methods and results of a health impact assessment of TransForm Baltimore, a rezoning effort in Baltimore, Maryland, and highlight findings specific to physical activity, violent crime and obesity	Mixed-methods study using secondary data analysis, policy documents review, and in-depth interviews with policy-makers from Department of Planning and city officials	Content analysis for qualitative data and quantitative impact assessment using ArcGIS (Esri, Redlands, USA)
Prasad et al., 2013^76^	India, South-East Asia Region, upper middle	To document strategies employed under the National Rural Health Mission, evaluate their impacts on reducing inequities and propose the mission as a model to address inequities	Case study by data collection using secondary data analysis and policy document review	Qualitative analysis using descriptive approach
Shei, 2013^70^	Brazil, Region of the Americas, upper middle	To examine whether the implementation and expansion of the major antipoverty (conditional cash transfer) *Programa Bolsa Família* was associated with improved infant health	Quantitative study using secondary data analysis	Quantitative analysis using predictive or modelling analysis
Addy et al., 2014^27^	Canada, Region of the Americas, high	To highlight a case for how polycentric governance underlying the whole-of-society approach is already functioning, while outlining an agenda to enable adaptive learning for improving such governance processes	Case study using secondary data analysis and policy document review	Qualitative data analysis using descriptive approach
Bardosh et al., 2014^39^	Lao People's Democratic Republic, Western Pacific Region, lower middle	To identify and investigate the sociocultural drivers and major transmission pathways of *Taenia solium*, assess community responses to an intervention, and explore locally acceptable strategies for long-term sustainable parasite control in the villages of highest incidence	Qualitative study using observation, semi-structured interviews and focus group discussion with community members	Thematic analysis for qualitative data
Baum et al., 2014^35^	Australia, Western Pacific Region, high	To determine the extent to which health in all policies is effective as a method of developing and delivering public policy that modifies the determinants of health in ways that improve population health and/or reduce health inequalities	Mixed-methods study using policy document review, semi-structured interviews, quantitative survey and focus group discussion with policy-makers	Thematic analysis for qualitative data and descriptive analysis for quantitative data
Bohn et al., 2014^69^	Brazil, Region of the Americas, upper middle	To verify whether conditional cash transfer policies have any impact on three important spheres of an individual’s life: consumption (the attainment of food security), inversion (access to the education system and acquisition of professional qualification) and production (entry into the job market)	Mixed-methods study using in-depth interviews and quantitative survey with community members	Content analysis for qualitative data and descriptive and inferential analysis for quantitative data
Nascimento et al., 2014^51^	Brazil, Region of the Americas, upper middle	To determine how social agendas are impacting living conditions and health in municipalities of the five regions of Brazil, and to demonstrate the impact of social agendas on selected millennium development goal indicators in Brazilian municipalities	Quantitative study using semi-structured interviews with municipal managers and secondary data analysis for the ecological longitudinal approach	Quantitative analysis using descriptive and inferential analysis
Nery et al., 2014^43^	Brazil, Region of the Americas, upper middle	To evaluate the impact of the *Programa Bolsa Família* and a family health programme on the incidence and detection of leprosy in Brazil during 2004–2011	Quantitative ecological study using secondary data analysis	Quantitative analysis using predictive or modelling analysis
Newman et al., 2014^26^	Australia, Western Pacific Region, high	To develop the evidence framework for healthy weight policy levers, develop a document analysis process, identify policy opportunities in South Australia government departments and consult with departments to develop policy recommendations	Qualitative study using policy document review	Qualitative analysis using descriptive approach
Pridmore et al., 2015^48^	Chile, Region of the Americas, high; Kenya, African Region, low	To use a controlled action research intervention and evaluate its impact on the nutritional status of children living in informal settlements in the cities of Mombasa (Kenya) and Valparaiso (Chile)	Non-RCT using quantitative survey and workshops involving policy-makers, health providers, community members and NGO representatives	Content analysis for qualitative data, and descriptive and inferential analysis for quantitative data
Kusuma et al., 2016^55^	Indonesia, South-East Asia Region, upper middle	To provide evidence on the effects of household and community cash transfers on determinants of maternal mortality, and provide a comparison of their effectiveness	RCT study using secondary data with clustered-randomized trials design	Quantitative analysis using inferential analysis
Olu et al., 2016^47^	African countries, African Region, low	To evaluate progress in the nine Sendai Framework for Disaster Risk Reduction targets, document lessons learnt and propose recommendations for accelerating the framework implementation within the health sectors	Mixed-methods study by using secondary data analysis, quantitative survey, and focus group discussion meetings involving health ministry policy-makers and NGO representatives (WHO)	Qualitative data analysis and descriptive analysis for quantitative data
Owusu-Addo, 2016^61^	Ghana, African Region, low	To understand the impact of conditional cash transfers on child health in rural Ghana	Qualitative study using semi-structured interviews with health providers, community members and programme implementers	Thematic analysis for qualitative data
Basso et al., 2017^50^	Uruguay, Region of the Americas, high	To assess the effectiveness of the Innovative Intervention approach and its acceptance	RCT study using entomological survey and quantitative survey with policy-makers and community members	Quantitative analysis using descriptive, inferential and cost analysis
Baum et al., 2017^40^	Australia, Western Pacific Region, high	To describe the extent to which non-health actors engaged with the South Australian health in all policies initiative, determine why they were prepared to do so and explain the mechanisms of successful engagement	Qualitative study using policy document review and in-depth interviews with policy-makers, academics and public servants	Thematic analysis for qualitative data
Durovni et al., 2017^52^	Brazil, Region of the Americas, upper middle	To examine the effect of the family health strategy and conditional cash transfer programme on tuberculosis outcomes in Rio de Janeiro	Secondary data analysis using data from patients in data registry	Quantitative analysis using inferential analysis
Ekirapa-Kiracho et al., 2017^53^	Uganda, African Region, low	To determine the effect of this participatory multisectoral intervention on the use of maternal and newborn services and care practices in the intervention and comparison areas, and determine the predictors of maternal service use and newborn care practices	Non-RCT using quasi-experimental pre- and post-comparison approach via observation of health provider or facility	Quantitative analysis using inferential analysis
Kananura et al., 2017^73^	Uganda, African Region, low	To explore the effect of a participatory multisectoral maternal and newborn intervention on birth preparedness and knowledge of obstetric danger signs among women in eastern Uganda	RCT study using quasi-experimental pre- and post-comparison design with health provider	Quantitative analysis using inferential analysis
Nery et al., 2017^44^	Brazil, Region of the Americas, upper middle	To evaluate the impact of the *Programa Bolsa Família* on the incidence of tuberculosis	Quantitative ecological study using secondary data analysis	Quantitative analysis using predictive or modelling analysis
Ruducha et al., 2017^57^	Ethiopia, African Region, low	To assess changes in the health and non-health policy and programme environment that contributed to or detracted from progress in child survival; examine the trends of health financing; assess coverage trends and equity of high-impact interventions; and develop estimates of selected high-impact interventions that possibly contributed to child survival using the Lives Saved Tool	Case study using secondary data analysis, policy document review, and in-depth interviews with policy-makers and NGOs	Descriptive analysis and predictive modelling using the Lives Saved Tool for quantitative data and evaluation framework from Countdown case study^82^
Triyana et al., 2017^58^	Indonesia, South-East Asia Region, upper middle	To extend earlier reports by exploring antenatal care component coverage for specific service items and antenatal care provider quality of midwives, and add to the current understanding on how conditional cash transfer programmes affect antenatal care services as a channel to improve pregnancy outcomes	Quantitative study using secondary data analysis	Quantitative analysis using descriptive and inferential analysis
Das et al., 2018^23^	Afghanistan, Eastern Mediterranean Region, low	To examine the effect of multisectoral collaboration using the case study of the Basic Package of Health Services	Case study by using secondary data analysis, policy document review, and focus group discussion involving policy-makers, health providers, NGO representatives and donors	Qualitative analysis using descriptive approach and content analysis
Hall et al., 2018^34^	USA, Region of the Americas, high	To explore whether government officials and advocates use the health in all policies framework to elevate health equity as a policy concern across sectors and jurisdictions	Qualitative study using semi-structured interviews with policy-makers and government officials	Thematic analysis for qualitative data
Milman et al., 2018^22^	Chile, Region of the Americas, high	To summarize progress towards implementation of *Chile Crece Contigo*, investigating how cross-sectoral collaboration and coordination were managed to provide integrated child development care on a national scale	Qualitative study using in-depth interviews and focus group discussion (multistakeholder dialogue) with policy-makers and health provider or facility	Thematic analysis for qualitative data
Renner et al., 2018^21^	Germany, European Region, high	To describe to what extent the change framework condition is reflected in the attitude and action of health actors and whether related to intersectoral changes, and identify barriers and facilitators for intersectoral collaboration	Mixed-methods study using guided telephone interview, expert focus groups and workshop with specialists, as well as monitoring survey	Descriptive and inferential statistics for quantitative data; qualitative data not presented
Sohn et al., 2018^31^	USA, Region of the Americas, high	To identify perceived effect of health impact assessments, and outline the mechanisms through which these effects can occur	Mixed-methods study using semi-structured interviews and quantitative survey with policy-makers, health providers, community members, private actors and NGOs	Thematic analysis for qualitative data, and descriptive and inferential analysis for quantitative data
Velásquez et al., 2018^32^	Guatemala, Region of the Americas, upper middle	To examine the factors that enable multisectoral collaboration	Case study using in-depth interviews with policy-makers, health providers, NGOs and donors	Thematic analysis for qualitative data
Agbo et al., 2019^33^	Guinea, Liberia and Sierra Leone, African Region, low	To outline the process of and highlight progress towards One Health institutionalization	Case study using secondary data analysis	Qualitative data analysis using descriptive approach
Baum et al., 2019^80^	Australia, Western Pacific Region, high	To examine the extent to which the activities of the South Australian health in all policies initiative can be linked to population health outcomes	Mixed-methods study using policy document review, semi-structured interviews and quantitative survey with policy-makers	Thematic analysis for qualitative data and inferential analysis for quantitative data
Hall et al., 2019^78^	Timor-Leste, South-East Asia Region, low	To investigate intersectoral collaboration for people-centred mental health care in the mental health system	Qualitative study using in-depth interviews with policy-makers, health providers, community members, private actors and NGOs	Qualitative data analysis using social network analysis
Kietzman et al., 2019^29^	USA, Region of the Americas, high	To describe collaborative efforts of Healthy Aging Partnerships in Prevention Initiative to enhance local capacity by training personnel from community health centres and community-based organizations, implementing a small grants programme and forming a community advisory council	Case study using situation report based on pilot study	Qualitative data analysis using descriptive approach
Moncayo et al., 2019^42^	Ecuador, Region of the Americas, lower middle	To evaluate the effect of social programme *Bono de Desarrollo Humano* in mortality of children < 5 years in counties (from poverty-related causes including diarrhoea, malnutrition and lower respiratory infections) and on some of the potential intermediate mechanisms	Quantitative ecological study using secondary data analysis	Quantitative analysis using predictive or modelling analysis
Olney et al., 2019^45^	Burundi, African Region, low	To estimate the secondary impacts of a food-assisted multisectoral nutrition programme (Tubaramure) on children’s motor and language development	RCT study using quantitative household survey and measurement of clinical indicators with community members (mother and children)	Quantitative analysis using descriptive, inferential, and predictive or modelling analysis
Pescud et al., 2019^28^	Australia, Western Pacific Region, high	To explore the public policy attention given to inequities in obesity using a case study	Qualitative study using in-depth interviews with policy-makers, government actors and NGO representatives	Thematic analysis for qualitative data
van Eyk et al., 2019^37^	Australia, Western Pacific Region, high	To provide insight into the facilitators of and impediments to intersectoral efforts to progress shared educational and health goals and achieve sustainable change, and identify lessons for others intending to use this approach	Mixed-methods study using secondary data analysis, policy document review and semi-structured interviews with policy-makers	Thematic analysis for qualitative data and descriptive analysis for quantitative data
Aizawa, 2020^77^	India, South-East Asia Region, lower middle	To describe the extent to which expanded eligibility criteria and increased cash incentive affect health care use, and to examine whether policy reform mitigates or deteriorates socioeconomic inequality in use of health care	Quantitative study using secondary data analysis	Quantitative analysis using descriptive and inferential analysis
de Araujo Palmeira et al., 2020^67^	Brazil, Region of the Americas, upper middle	To examine prospectively access to 27 government programmes related to food and nutrition services among families living in a socioeconomically deprived municipality during 2011–2014, and determine whether access to different programmes was associated with changes in the household food insecurity status over time	Quantitative study using cross-sectional survey with community members and policy document review	Quantitative analysis using descriptive and inferential analysis
Stoner et al., 2020^62^	South Africa, African Region, upper middle	To determine how the cash transfer intervention (*Swa Koteka*) and components of study participation influenced sexual behaviour in young women (age 13–20 years), and explore mechanisms through which the programme affected this behaviour	Qualitative study using semi-structured interviews with community member (young women)	Thematic analysis for qualitative data
Alves et al., 2021^65^	Brazil, Region of the Americas, upper middle	To investigate the association between the expansion of the *Programa Bolsa Família* in Brazil and malaria incidence in endemic Brazilian municipalities between 2004 and 2015	Quantitative ecological study using secondary data analysis	Quantitative analysis using descriptive and inferential analysis
Asaaga et al., 2021^41^	India, South-East Asia Region, lower middle	To inform the effective operationalization of contextually appropriate One Health by improving practical understanding of the policy and local influences its implementation, and identify barriers and facilitators linked to the prevention and control of zoonoses	Qualitative study using policy document review and semi-structured interviews with key actors and One Health practitioners	Thematic analysis for qualitative data
Ramponi et al., 2021^81^	Malawi, African Region, low	To illustrate an analytical framework that lays out the various effects, and makes explicit the opportunity costs, of a social cash transfer programme to each stakeholder to communicate the value of a cross-sectoral policy	Quantitative economic evaluation study using secondary data analysis	Quantitative analysis using predictive or modelling analysis
Rasella et al., 2021^30^	Brazil, Region of the Americas, upper middle	To assess the impact of the *Programa Bolsa Família* on maternal mortality and evaluate its effects on potential intermediate mechanism	Quantitative study using secondary data analysis	Quantitative analysis using predictive or modelling analysis
Turner et al., 2021^74^	Colombia, Region of the Americas, upper middle	To analyse how intersectoral coordination took place in three cities (Bogota, Cali and Cartagena) and describe the main roles that two sectors (academic institutions and private enterprise) assumed in their efforts to assist the response of the health sector to the COVID-19 pandemic	Qualitative study using semi-structured interviews with policy-makers, private actors and academia	Thematic analysis for qualitative data
Al Dahdah et al., 2022^49^	India, South-East Asia Region, lower middle	To explore the genesis of India’s digital turn in health care and map the characteristics of such a policy, based on empirical analysis of *Rashtriya Swasthya Bima Yojana*, India’s first digital-based UHC programme	Qualitative study using secondary data analysis and in-depth interviews with policy-makers, health providers, community members and private actors	Thematic analysis for qualitative data
Blanken et al., 2022^36^	Netherlands (Kingdom of the), European Region, high	To explore and compare the development of structures of information exchange in networks over time, concerning both material and knowledge-based information	Mixed-methods study using semi-structured interviews and quantitative survey with policy-makers, health providers, community members and NGOs	Descriptive analysis for quantitative data and social network analysis
Bokhour et al., 2022^56^	USA, Region of the Americas, high	To evaluate the use of a whole health system of care on opioid use (because of the focus of the Comprehensive Addiction and Recovery Act focus on opioid use) and assess the impact on patient-reported outcomes	Quantitative case–control study using secondary data analysis and quantitative survey with community members (veterans)	Quantitative analysis using descriptive and inferential analysis
Machado et al., 2022^68^	Brazil, Region of the Americas, upper middle	To investigate the association of a large conditional cash transfer programme with the reduced occurrence of suicide	Non-RCT using quasi-experimental pre- and post-comparison using secondary data analysis	Quantitative analysis using inferential analysis
Wang et al., 2022^59^	China, Western Pacific Region, upper middle	To investigate China’s COVID-19 vaccination system and summarize its implementation experience from a health system perspective	Qualitative study using policy document review and semi-structured interviews with policy-makers, health provider and government staff at community level	Thematic analysis for qualitative data
de Jong et al., 2023^24^	Netherlands (Kingdom of the), European Region, high	To provide insights into the processes of a coalition that facilitate building and maintaining intersectoral collaboration within a health promotion programme, and describe how these processes contribute to the success of the coalition	Qualitative study using in-depth interviews and observation with community members and private actors	Qualitative analysis using document and composed network analysis
Jimenez et al., 2023^79^	Ethiopia and countries in western Africa, African Region, low	To describe how the bottom-up community inclusiveness developed during the Ebola virus disease outbreak enhanced pandemic preparedness, and how community resilience was improved through sustainable entrepreneurs implementing One Health policies	Case study using participant observation and policy document review	Qualitative analysis using descriptive approach
Naughton et al., 2023^75^	Ireland, European Region, high	To explore the experiences of the members of the schools teams model in Ireland to identify factors that influenced effective interdisciplinary working, and describe how lessons learnt can inform future multisectoral collaborations to address complex public health priorities	Mixed-methods study using semi-structured interviews and online survey with schools teams members	Thematic analysis for qualitative data and descriptive analysis for quantitative data
Sello et al., 2023^63^	South Africa, African Region, upper middle	To identify how different care support systems can be linked to ensure optimal childhood nutrition outcomes	A sequential mixed-methods approach	Descriptive quantitative analysis and thematic analysis for qualitative data
Silva et al., 2023^64^	Brazil, Region of the Americas, upper middle	To characterize the nutritional and breastfeeding status of children < 2 years among both beneficiaries and non-beneficiaries of *Programa Bolsa Família*	A cross-sectional study based on food and nutritional surveillance data	Quantitative data analysis using χ^2^ and estimating odds ratio

COVID-19: coronavirus disease 2019; NGO: nongovernmental organization; RCT: randomized controlled trial; UHC: universal health coverage; WHO: World Health Organization.

^a^ World Bank Classification.

**Table 2:** Distribution of studies included in a systematic review of the effect of multisectoral interventions for health on health system performance, according to WHO region and design

Characteristics	No. of studies (%) (*n* = 62)
**WHO region**	
African Region	12 (19.4)
Region of the Americas	27 (43.5)
South-East Asia Region	7 (11.3)
European Region	6 (9.7)
Eastern Mediterranean Region	1 (1.6)
Western Pacific Region	8 (12.9)
Multiple regions	1 (1.6)
**Income level (World Bank classification)**	
High	22 (35.5)
Upper middle	23 (37.1)
Lower middle	5 (8.1)
Low	11 (17.7)
Multiple countries of different income levels	1 (1.6)
**Primary data collection strategies^a^**	
Secondary data analysis	28 (45.2)
Semi-structured or in-depth interviews	28 (45.2)
Quantitative surveys	17 (27.4)
Policy document analysis	16 (25.8)
Focus group discussion or workshop	10 (16.1)
Observation	4 (6.5)
**Primary data analysis methods^a^**	
Quantitative data analysis (e.g. descriptive, inferential and predictive)	32 (51.6)
Qualitative analysis (both thematic and content)	29 (46.8)
Mixed analysis	10 (16.1)
Social network analysis	3 (4.8)

WHO: World Health Organization.

^a^ Some studies may have used more than one data collection or analysis method.

We observe that the reviewed studies employed a variety of study designs, with the largest proportions using quantitative (30.6%; 19 studies), qualitative (24.2%; 15 studies) and mixed (21.0%; 13 studies) methods. A small number of publications described randomized controlled trials (RCTs), non-RCT designs and case study methods. The largest proportion of studies focused on multisectoral interventions directly related to specific health outcomes (66.1%; 41 studies) and/or social determinants of health (48.4%; 30 studies) without explicit reference to overall health system performance. We provide more details on data collection and analysis methods in Table 3.

**Table 3:** Characteristics of multisectoral collaborations described in systematic review of the effect of multisectoral interventions for health on health system performance

Characteristic	No. of studies (%) (*n* = 62)
**Type of collaboration^a^**	
Joined-up government (health and non-health sectors)	10 (16.1)
Health in all policies or whole-of-government approach	7 (11.3)
Integrated health and social services (including poverty reduction)	17 (27.4)
Collaborative governance	4 (6.5)
Social determinants of health and sustainable development	8 (12.9)
Public and private partnership	4 (6.5)
Formal and informal partnership	4 (6.5)
Health impact assessment	2 (3.2)
Policy and/or community networks	1 (1.6)
Collaboration on specific issues: One Health and zoonosis	5 (8.1)
Collaboration on specific issues: maternal and child health	13 (21.0)
Collaboration on specific issues: mental health	4 (6.5)
**Sector involvement^a^**	
Health sector (including health facilities and providers)	62 (100.0)
Non-health government sector (e.g. education, agriculture, water and environment, social and welfare, transportation or telecommunication)	57 (91.9)
Nongovernmental organization	14 (22.6)
Informal sector	3 (4.8)
Community organization	15 (24.2)
Academia or university	8 (12.9)
International bodies	4 (6.5)
Donor agency	4 (6.5)
Private sector	4 (6.5)
Police department or security	2 (3.2)
**Indicators of collaboration**	
Yes	10 (16.1)
**Sustainability issues**	
Yes	17 (27.4)

^a^ Some studies may be of more than one collaboration type; all studies involve multiple sectors.

### Characteristics of multisectoral collaborations

In Table 3 we list the characteristics of the multisectoral collaborations described in the reviewed publications, including types of collaboration and sector involvement. The studies reported on various key objectives of multisectoral collaborations for health, which we attempted to categorize into five themes as far as possible (Box 2); not all studies could be categorized as a single theme or, in some cases, any of the themes.

Box 2 **Key categories of multisectoral collaborations studied in systematic review of the effect of multisectoral interventions for health on health system performance**

1. Improving cross-collaboration between ministries or government departments to enhance health, social and education services;^22^^,^^28^^,^^33^^,^^38^^–^^40^
2. promoting the effectiveness of governance;^20^^,^^33^^,^^41^
3. enhancing access to health services, population health outcomes and reducing health and/or social inequities;^21^^–^^25^^,^^32^^,^^35^^,^^36^^,^^42^^–^^64^
4. providing evidence-based strategies and policy recommendations to address social determinants of health and mutual goals across government sectors;^26^^,^^30^^,^^47^^,^^65^^–^^74^ and
5. strengthening programme implementation.^29^^,^^37^^,^^47^^,^^75^^–^^81^

Most (83.9%; 52) of reviewed publications did not address process indicators; only 10 studies provided such descriptions. The process indicators addressed included improved access to multisector services through social protection programmes; fund transfer agreements for quality and accountability; integrated monitoring and evaluation;^22^ or the importance of strengthening relationships between government agencies to address child nutrition issues.^23^ Others advocated measures of suitability of partners, functioning of the coalition, agreement about mission or perceived interpersonal relations between coalition members;^24^ or the active involvement of partners.^25^^,^^40^ One study proposed that a strong indicator for a successful collaboration is an increased perceived importance of intersectoral collaboration (in this case, health in all policies).^25^Other studies included other indicators: fostering collaboration among One Health stakeholders and increasing One Health advocacy activities;^33^ enhancing collaboration among actors to address neglected tropical diseases and improving integrated actions;^39^ improving cross-sector engagement;^41^ building capacity across sectors;^53^ and strengthening network relationships.^78^

A large proportion (72.6%; 45) of reviewed publications did not address or discuss sustainability for multisectoral interventions in health. Some authors proposed sustainability mechanisms, including strengthening government commitment to multisectoral approaches;^26^ promoting good governance practices, community participation and capacity-building;^24^^,^^27^^,^^28^ and institutionalization of the intervention with increased budget allocation from the national government.^22^^,^^29^^–^^32^ Other strategies involved strengthening national ownership along with donor investment and cooperation,^33^^–^^35^ sustaining network managers and public officials,^36^ and promoting the involvement of volunteer labour.^37^

### Effects on health system performance

Although most studies were not designed to assess the impacts of multisectoral interventions on overall health system performance, many addressed partial, more proximate components of health system functions that were perceived as directly related effects. Crucially, none of the included studies explicitly incorporated health system design (from building blocks to health outcomes) when attributing observed effects on health system performance to multisectoral collaborations. We provide a summary of the effects of multisectoral approaches on health system performance, as described by included studies and guided by the WHO framework for health system performance assessment,^83^ in Table 4. From the intermediate perspective, most studies (80.6%; 50) focused on the service delivery function of health systems or on environments that enabled access to care. We provide some examples of these effects (intermediate and final or ultimate goals) in Box 3.

**Table 4:** Effects on health system performance noted in systematic review of multisectoral interventions for health

Description of effects	No. of studies (%) (*n* = 62)
**Intermediate objective: access and service delivery** ^a^	
Improved access to health services, such as screening for early developmental delay, preventive measures, maternal and child health services, mental health services	18 (29.0)
Improved collaboration across health services and delivery	7 (11.3)
Improved service availability and readiness for addressing zoonotic diseases, enhanced staff skills in the provision of maternal and child health, pandemic preparedness	6 (9.7)
Improved acceptability of services	8 (12.9)
Improved affordability of services	8 (12.9)
Improved adequacy of funding	3 (4.8)
Improving safety and quality of health services	1 (1.6)
Improved efficiency of service	1 (1.6)
**Intermediate objective: enabling environment for promoting access to services^a^**	
Improved enabling of environments for health (e.g. improved social economic conditions, improved Gini Index, school enrolments, increased productivity, stable family income, food security, addressing maternal health determinants)	25 (40.3)
Strengthening support systems for health by leveraging expertise and capacity from allied sectors, commitment from stakeholders for health, policy processes that support health	16 (25.8)
**Ultimate health system goals^a^**	
Improved access equity for developmental screening, other health services (tuberculosis, nutrition, vaccination, access to healthy food, social equity), addressing barriers of a low-resource setting, allowing equitable access for mental health care	28 (45.2)
Improved health outcomes such as treatment success for developmental disorders, reduced hospitalization or mortality, reduced morbidity (from malnutrition or infections, tuberculosis incidence), improved quality of life from ministerial perspective (number of disability-adjusted life years averted), maternal mortality, tuberculosis treatment compliance	26 (41.9)
Improving fair financing and financial risk protection for vulnerable populations (e.g. reducing out-of-pocket payments for rural communities)	1 (1.6)
Supporting community participation and/or capacity (e.g. for maternal and child health services, mental health care, co-design or bottom-up approaches)	8 (12.9)
Reported harms or unintended consequences such as increasing rural and urban digital health divide, reduced economic benefit from donor’s perspective, bureaucratic barriers because of multiple governance levels	3 (4.8)

^a^ Some studies may have more than one objective or health system goal.

Box 3 **Examples of effects of reviewed multisectoral interventions for health on intermediate and ultimate goals of health systems**

1. An impact evaluation of a food-assisted maternal and child health and nutrition programme (*Tubaramure*) targeting Burundian women and children found that, using language and motor developments as indicators, the first 1000 days of the programme positively affected health outcomes of children.^45^
2. An impact evaluation of the Nutritional Improvement for Children in Urban Chile and Kenya (NICK) intervention, involving various government agencies including health, education, water, agriculture and social development sectors, along with many local stakeholders, found that the programme reduced child stunting.^48^
3. An intersectoral ecosystem management intervention with and without community participation in Uruguay, involving health ministry, social development ministry, community, and local government and stakeholders, reported reduced vector densities in intervention clusters (i.e. decreased in the intervention clusters 11 times and in the control clusters only four times). The programme also promoted community acceptability and participation. A cost analysis of the programme found that the costs of the intervention activities in the scaling-up process (without community participation) were 45.6% lower compared with the estimated costs of the routine activities executed by the health ministry and the Salto municipality.^50^
4. The maternal and neonatal implementation for equitable system (MANIFEST) project was implemented in three rural Ugandan districts using a participatory multisectoral intervention to improve utilization of maternal and newborn services and care practices. The intervention increased: early antenatal clinic attendance by 8% and facility delivery by 7%; improved clean cord care by 20%; and delayed bathing by 8%.^53^ Additionally, the project improved the birth preparedness practices and knowledge of obstetric danger signs, critical for improving maternal services utilization.^73^
5. A quasi-experimental study compared a group who participated in a cash transfer intervention (*Programa Bolsa Família*) with those who did not. The study found that beneficiaries had lower suicide rate than non-beneficiaries. The intervention could possibly help to prevent suicide by intervening in factors related to poverty, which can lead to suicide.^68^
6. An impact evaluation of household cash transfers and community cash transfers on determinants of maternal mortality in Indonesia found that community cash transfers had a more positive impact on determinants such as maternal health knowledge, financial barriers, utilization among higher-risk women, *Posyandu* (integrated health post) equipment and nutritional intake. The effects of household cash transfers were only observed in utilization of health services.^55^

### Intermediate objectives

Many of the reviewed publications focused on improving access to care,^22^^,^^27^^,^^29^^–^^32^^,^^43^^,^^53^^,^^55^^,^^58^^,^^62^^,^^65^^,^^69^^,^^70^^,^^73^^,^^76^^–^^78^ service delivery,^22^^,^^32^^,^^45^^,^^52^^,^^53^^,^^57^^,^^74^ affordability,^27^^,^^30^^,^^57^^,^^62^^,^^67^^–^^69^^,^^76^ acceptability,^30^^,^^32^^,^^56^^,^^65^^,^^69^^,^^70^^,^^77^^,^^79^ and service readiness and availability.^41^^,^^45^^,^^53^^,^^59^^,^^74^^,^^79^ Other indicators such as improving efficiency of services,^29^ adequacy of funding,^30^^,^^57^^,^^69^ and safety and quality of health services^30^ were only studied in a small number of publications; cost and productivity, and administrative efficiency, were not discussed in any of the reviewed publications. The selection of short-term outcome indicators was closely related to the nature of interventions. For instance, many papers focused on conditional cash transfers with mandatory school enrolment and health attendance, allowing families to afford health services.^42^^–^^44^^,^^46^^,^^58^^,^^61^^,^^65^^,^^69^ Similarly, studies addressing specific issues such as maternal and child health,^23^^,^^53^^,^^73^^,^^77^ One Health or zoonotic diseases,^33^^,^^39^^,^^41^^,^^79^ and mental health^71^^,^^78^ contributed to health system preparedness, resulting in improved acceptability, availability and readiness. Interventions aimed at enhancing the skills of health workers in providing maternal and child services were found to improve leadership skills, fostering a more efficient and effective environment for delivering maternal health services.^53^

Reviewed publications also focused strongly on examining enabling environments for health,^26^^–^^28^^,^^30^^–^^32^^,^^35^^,^^38^^,^^40^^,^^45^^,^^48^^, ^^50^^,^^54^^,^^55^^,^^57^^,^^61^^,^^66^^,^^67^^,^^69^^–^^72^^,^^76^^,^^80^^,^^81^ strengthening support systems for health^24^^,^^27^^,^^28^^,^^33^^,^^37^^,^^38^^,^^41^^,^^47^^,^^51^^,^^52^^,^^61^^,^^67^^,^^68^^,^^74^^–^^76^ and community participation.^31^^,^^37^^–^^39^^,^^48^^,^^53^^,^^78^^,^^79^ These studies underscored the pivotal role of non-health sectors or actors in reducing access barriers to health services and preventive health measures by tackling social determinants of health.^26^^,^^27^^,^^45^^,^^66^^,^^80^ Active participation of non-health actors in addressing health issues can provide a fertile foundation for resource sharing and health programme implementation, as seen in health preparedness for disasters.^47^ Collaborations around zoonotic diseases also facilitated mutual interest across government agencies, strengthening the supportive environment for health interventions.^33^ Attention to the enabling environment for health emerged as a crucial aspect, with multisectoral efforts contributing to the development of policies and frameworks that promote health and well-being.

### Effects on ultimate health system goals

Most of the reviewed publications (66.1%; 41) considered health system goals. Of these studies, the majority focused on improving health equity (68.3%; 28) and health outcomes (63.4%; 26). A small number of studies explored patient centredness,^23^^,^^32^^,^^53^^,^^56^^,^^62^^,^^71^^,^^74^^,^^78^ or fair financing or financial risk protection.^76^ No studies reported on satisfaction levels for patients or health providers. The single publication addressing financial risk protection was conducted in India, exploring the implementation of the National Rural Health Mission to address social determinants of health and strengthen health systems.^76^ This case study found that the mission reduced mortality rates for both infants and mothers, bridging inequities between urban and rural settings, and decreasing out-of-pocket payments for rural communities.^76^ Collaborations between health and non-health sectors play a pivotal role in promoting health and social equities. By addressing the social determinants of health, these interventions contribute to a more equitable distribution of health-care resources and outcomes. Concurrently, improvements in overall health outcomes signify the enduring success of multisectoral interventions, reflecting a holistic and sustained approach to health system performance.

### Potential unintended consequences

Three studies reported potential unintended consequences from multisectoral interventions for health.^37^^,^^49^^,^^81^ The implementation of digital health for all in India created barriers to accessing digital health services, particularly for people residing in rural settings and poor families,^49^ further exacerbating the digital health divide between affluent and poorer areas. An economic evaluation of a social cash transfer programme in Malawi found that, although the intervention brought economic benefits from the government perspective (increased total number of averted disability-adjusted life years), it offered less economic value for donors who were more inclined to invest in disease-specific models rather than social cash transfer programmes.^81^ Various governance models for multisectoral interventions can also create confusion and bureaucratic barriers before implementation of system-wide strategies, thereby delaying well-intended health programmes.^37^

### Potential mechanisms

Of the included publications, 40 studies (64.5%) proposed mechanisms explaining how multisectoral interventions for health could lead to the intended outcomes, such as improved access to health services, promotion of health equity and improved health outcomes. The reviewed publications referred to collaborative participation and engagement of various frontline actors,^23^^,^^27^^,^^28^^,^^30^^–^^32^^,^^36^^,^^37^^,^^48^^,^^50^^,^^53^^, ^^56^^,^^57^^,^^59^^,^^65^^,^^66^^,^^70^^,^^71^^,^^74^^,^^76^^,^^77^ collaborative leadership and governance,^22^^,^^24^^–^^29^^,^^33^^,^^35^^,^^37^^,^^40^^,^^48^^,^^57^^,^^59^^,^^76^^,^^79^^,^^80^ governance arrangements,^23^^,^^27^^,^^29^^,^^33^^,^^37^^,^^39^^,^^40^^,^^54^^,^^78^^,^^79^ and informed sectors or actors^26^^,^^27^^,^^37^^,^^40^^,^^71^^,^^74^^,^^75^^,^^81^ as possible mechanisms. Only five publications acknowledged power dynamics or relations as having an explanatory effect.^27^^,^^32^^,^^33^^,^^40^^,^^76^

## Discussion

Our systematic review contributes a comprehensive understanding of the current state of knowledge regarding multisectoral interventions and their impact on health system performance. We have described how multisectoral interventions can promote robust health system performance, yet also highlighted how many of these effects remain assumed rather than substantiated. Reviewed publications have demonstrated that multisectoral health interventions can enable integrated service models by fostering partnerships between health and non-health sectors, streamlining service delivery and enhancing coordinated care for target populations. 

We identified key types of collaboration, but found little emphasis on process measures, sustainability or potential harms. We also found limited assessment of overall health system performance goals, with assumptions about generalized effectiveness and a focus on measurement of proximate and intermediate outcomes. We noted a relative emphasis on speculative mechanisms of effect, but little direct evidence.

Previous studies have provided similar descriptions of features of multisectoral interventions that enhance acceptability and affordability of health services, such as cross-sectoral training, resource sharing and joint planning.^15^ Involving non-health sectors allows for diverse community participation, addresses social determinants and financial barriers, advocates improved health outcomes and enhances the overall health system readiness to address emerging challenges.^84^^,^^85^ Collaboration across sectors provides opportunities for integrated information systems, improving service delivery accuracy and efficiency for informed decision-making.^86^^,^^87^ A review examining the effects of multisectoral collaboration on health and well-being also found improvements in service delivery, efficiency and effectiveness, but limited evidence for change in health outcomes.^88^ Others have also speculated that by reducing barriers between health and non-health sectors, multisectoral collaborations streamline service delivery mechanisms, ensuring that resource utilization is increased and optimized.^9^^,^^10^^,^^89^

In our reviewed publications, we noted a common theme of the facilitation of community participation. Multisectoral interventions empower communities to engage in their health and well-being^85^ by breaking down barriers between sectors and taking an active role in shaping their health outcomes.^90^^,^^91^ This approach contributes to immediate improvements in service acceptability and fosters a sense of ownership and agency among community members. Community participation becomes a driving force behind the sustained success of multisectoral interventions, enhancing health system performance over time.^85^

Fundamental to our findings is the recognition that building a robust health system necessitates collaborative efforts that transcend traditional health sector boundaries. The inclusion of non-health sectors is paramount in driving interventions that address the multifaceted determinants of health. This multisectoral approach acknowledges that health outcomes are not solely contingent upon medical interventions, but are profoundly influenced by social, economic and environmental factors.^1^^,^^84^^,^^89^ Fostering partnerships between health and non-health sectors is therefore imperative for comprehensive and effective health system performance.^2^^,^^92^ Consequently, our review underscores the imperative of the health sector to collaborate with diverse stakeholders, each wielding unique influence and power. For example, collaborative actions between health and education are crucial for community participation,^13^ and partnerships with the social and welfare sector can address financial barriers for accessing health services.^42^^,^^43^ These partnerships signal shared responsibility across sectors for promoting population health outcomes, challenging traditional silos in health interventions.^9^^,^^10^^,^^89^ Multisectoral collaboration for health is essential for health system strengthening to promote health improvement and equity.^15^^,^^85^

Our systematic review has some limitations. Although the geographic diversity of included studies suggests global interest in and relevance of such interventions, the predominance of studies from high- and upper-middle-income countries raises questions about the generalizability of findings to low-resource settings, and flags a potential research gap in understanding the dynamics of these interventions in low-income countries. Additionally, because of heterogeneity in the reviewed publications, as well as the complex nature of interventions and the broad range of possible effects, pooled synthesis is not always possible.

Our review highlights significant research gaps that warrant future investigation. The paucity of studies explicitly incorporating health system design suggests a possible conceptual gap and the need for a more holistic understanding of the effects of multisectoral collaborations on health system performance, at a range of measurement levels. Most papers lacked a systematic exploration of process indicators, and intermediate effects primarily targeted proximate outcomes. Relatively under-researched aspects of health system performance – such as cost and productivity, quality and safety, or unintended consequences – offer areas for further exploration and vigilance in response to implementation. We identified some differential effects for different actors within health systems; however, the lack of a realistic evaluation among the reviewed publications may highlight a theoretical gap in comprehensively exploring the contextual factors and mechanisms that contribute to the success or failure of multisectoral interventions.

To conclude, multisectoral interventions influence health system performance by improving service delivery efficiency, readiness, acceptability and affordability. Although multisectoral interventions for health can improve health equity and outcomes, evidence remains limited in relation to financial risk protection and satisfaction levels. The holistic benefits of these interventions underscore the essential role of multisectoral collaborations in addressing the complexities of modern health-care challenges and strengthening health systems through coordinated service delivery, healthy policies, and addressing social determinants and financial barriers.
